# The VEGF-Receptor Inhibitor Axitinib Impairs Dendritic Cell Phenotype and Function

**DOI:** 10.1371/journal.pone.0128897

**Published:** 2015-06-04

**Authors:** Annkristin Heine, Stefanie Andrea Erika Held, Solveig Nora Daecke, Kati Riethausen, Philipp Kotthoff, Chrystel Flores, Christian Kurts, Peter Brossart

**Affiliations:** 1 Department of Oncology, Hematology and Rheumatology, University Hospital Bonn, Bonn, Germany; 2 Institute of Experimental Immunology (IEI), University Bonn, Bonn, Germany; Istituto Superiore di Sanità, ITALY

## Abstract

Inhibitors of VEGF receptor (VEGFR) signaling such as sorafenib and sunitinib that are currently used in the treatment of malignant diseases have been shown to affect immunological responses by inhibition of the function of antigen presenting cells and T lymphocytes. The VEGFR-inhibitor axitinib has recently been approved for second line therapy of metastatic renal cell carcinoma. While there is some evidence that axitinib might interfere with the activation of T cells, not much is known about the effects of axitinib on dendritic cell (DC) phenotype and function. We here show that the addition of axitinib during the final Toll-like receptor-4-induced maturation step of monocyte-derived human DCs results in a reduced DC activation characterized by impaired expression of activation markers and co-stimulatory molecules such as CD80, CD83 and CD86. We further found a decreased secretion of interleukin-12 which was accompanied by reduced nuclear expression of the transcription factor cRel. In addition, we found a dose-dependent reduced activation of p38 and STAT3 in axitinib-exposed DCs, whereas the expression was not affected. The dysfunction of axitinib-exposed DCs was further underlined by their impaired induction of allogeneic T cell proliferation in a mixed lymphocyte reaction assay and inhibition of DC migration. Our results demonstrate that axitinib significantly affects DC differentiation and function primarily via the inhibition of the nuclear factor kappa B signaling pathway leading to impaired T cell activation. This will be of importance for the design of future vaccination protocols and therapeutic approaches aiming at combining different treatment strategies, eg such as programmed death-1 inhibitors with axitinib.

## Introduction

Renal cell cancer (RCC) hardly responds to conventional radio- or chemotherapy. However, spontaneous regression rates are higher than in other tumors and high infiltrations of immune cells are regularly found in RCC lesions[1]. This immunologic setting has yielded in the development of immunotherapeutic treatment regimens in RCC, such as the use of Interleukin-2 (IL-2) or peptide- and ribonucleic acid (RNA)-based vaccination protocols[1–3]. The approval of tyrosine kinase inhibitors (TKI) for the treatment of RCC has further changed the course of disease. Sunitinib and sorafenib are established first line therapies for metastatic RCC, whereas the vascular endothelial growth factor receptor (VEGFR)-inhibitor axitinib has been approved for second line therapy. All three compounds block VEGFR, axitinib most selective, and are known to possess anti-angiogenic, but also immune-modulatory functions[1]. Sorafenib and sunitinib both have been described to exert distinct, but different effects on immune cells. Sorafenib, but not sunitinib has immunosuppressive properties on dendritic cells (DCs)[4], whereas all three compounds have been described to decrease T cell proliferation[5]. The extended choice of therapy options led to the question which compounds can be combined and whether the use of some of these TKIs might interfere with simultaneous or sequential immunotherapeutic approaches.

Angiogenesis and immunosuppression are closely linked in the tumor microenvironment. While tumor growth is associated with impaired antitumor immune responses, VEGF is essential for tumor-induced angiogenesis, but also plays a major role in tumor-associated immunosuppression[6]. Of note, it is known that VEGF influences various immune cells, such as it alters the growth and maturation of immature granulocyte-macrophage progenitors, but can also prevent DC precursors from developing into mature, antigen-presenting DCs[6]. Furthermore, VEGF influences DC–endothelial cell cross-talk, DC trans-differentiation, and tumor-associated macrophage infiltration[6].

Since DCs are the most powerful antigen presenting cells (APC) and critical regulators orchestrating adaptive immune responses *in vivo*[7,8], inefficient DC activation is unfavorable for latter induction of efficient CD8^+^ T cell responses. In consequence, anti-tumor immune responses can be compromised resulting in enhanced tumor growth.

Therapies inhibiting VEGF or VEGFR have demonstrated improved anti-tumor immunity and enhanced responses to cancer vaccines[6]. Modulation of vessel modification might account for a relevant part of tumor regression in these studies, however it is not entirely clear whether VEGFR-inhibition also directly affects DC phenotype and function and might even counterbalance some of the induced positive anti-cancer effects.

Until now, potential effects of the clinically used VEGF-inhibitor axitinib on the development and functional activity of immune cells are not completely understood. We here establish a new role for axitinib as modulator of DC function, which in consequence impairs proper T cell activation.

## Methods

Buffy coats for human monocyte isolation were obtained from voluntary blood donors of the University Hospital Bonn. Approval was obtained from the Institutional Ethics Committee of the University of Bonn, North-Rhine Westphalia, Germany (grant number 173/09).

Written informed consent was obtained for blood donation and further processing of blood samples for scientific purposes by the blood bank/transfusion medicine of the University Hospital of Bonn.

### Media and reagents

Cells were cultured in RPMI 1640 containing glutamax-I, supplemented with 10% inactivated fetal calf serum (RP10 medium) and 1% penicillin/streptomycin (Invitrogen). Axitinib was obtained from Selleckchem. All reagents not otherwise indicated were purchased from Sigma-Aldrich.

### Generation of DCs

Human monocyte-derived DCs (moDCs) were generated from peripheral blood by plastic adherence as described previously[9–11]. Adherent monocytes were cultured in RP10 medium supplemented with GM-CSF (100 ng/mL; Leukine, Liquid Sargramostim) and IL-4 (20 ng/ml; R&D Systems). Cytokines were added to differentiate DCs every other day.

Axitinib was dissolved in dimethyl sulfoxide (DMSO) and added to the culture media in concentrations varying from 0.1 to 10 μM, which corresponds to serum levels achieved in treated patients. In each case, equal amounts of DMSO were added as a control.

### Immunostaining

MoDCs were stained using commercially available mAbs from BD Biosciences, Dako Diagnostika, Immunotech, R&D Systems and eBioscience.

### Determination of cytokine production

For cytokine analysis in cell culture supernatants, FlowCytomix Multiple Analyte Detection commercial assay kits were used (eBioscience) and Flow Cytomix Pro Software.

### *In vitro* migration assay

A total of 1x10^5^ cells were seeded into a transwell chamber (8 μm; BD Falcon) in a 24-well plate, and migration to CCL19/MIP-3β was analyzed after 4 h by counting gated DCs for 1 minute in a FACS cytometer.

### Mixed lymphocyte reactions

A variable number of irradiated stimulator DCs was cultured with a total of 1x10^5^ responding allogeneic peripheral blood mononuclear cells. Tritium-labeled thymidine incorporation was measured on day 5 by a 16-hour pulse with [^3^H]-thymidine (18.5 kBq/well; GE Healthcare).

### Detection of apoptosis

Apoptosis in DCs was detected by live-dead staining using the propidium iodide (PI) or 7AAD-annexin V staining kit from eBioscience.

### Polyacrylamide gel electrophoresis and Western blotting

Whole cell lysates were prepared as described previously[10]. Protein concentrations were determined using a bicinchoninic acid assay (Pierce, Perbio Science). For analysis of the activation and expression status of Caspase-3 (31A1067, purchased from Santa Cruz Biotechnology), 20 μg whole-cell lysates were separated on a polyacrylamide gel and transferred on a nitrocellulose membrane[12]. The blots were probed with monoclonal antibodies against pro-Caspase-3 as well as GAPDH (10B8, Santa Cruz Biotechnology) as loading control, with phosho-p38 (T180/Y182) and p38 (both purchased from Cellsignaling; expression was determined as loading control) or phospho-Stat3 (Y705, 3E2) and Stat3 (124H6, both purchased from Cellsignaling; expression was determined as loading control). Nuclear extracts from moDCs were prepared as described before[11]. For analysis of nuclear protein out of 10^6^ cells, 20 μl of nuclear extracts were separated on a 10% SDS-PAGE and transferred on a nitrocellulose membrane. Ponceau S staining of the membrane was performed to ensure that equal amounts of protein had been loaded onto the gel. Subsequently, the blot was probed with antibodies against RelA (D14E12, Cellsignaling), c-Rel, RelB (B-6 and C-19, both purchased from Santa Cruz Biotechnology) and HDAC1 as additional loading control (H-51, SantaCruz Biotechnology). Protein bands were detected using an enhanced chemiluminescence kit (GE Healthcare).

### Statistical analysis

All experiments were performed at least 3 times, with representative experiments shown. To analyze statistical significance, a Student´s t test was used. Comparisons were made as indicated with the Mann-Whitney test or one-way ANOVA and Dunnetts using Prism 5 software (Graphpad Software).

## Results

### Axitinib dose-dependently induces cell death in the renal cell carcinoma cell line A-498, but has no toxic effects on moDCs

**Fig 1:**
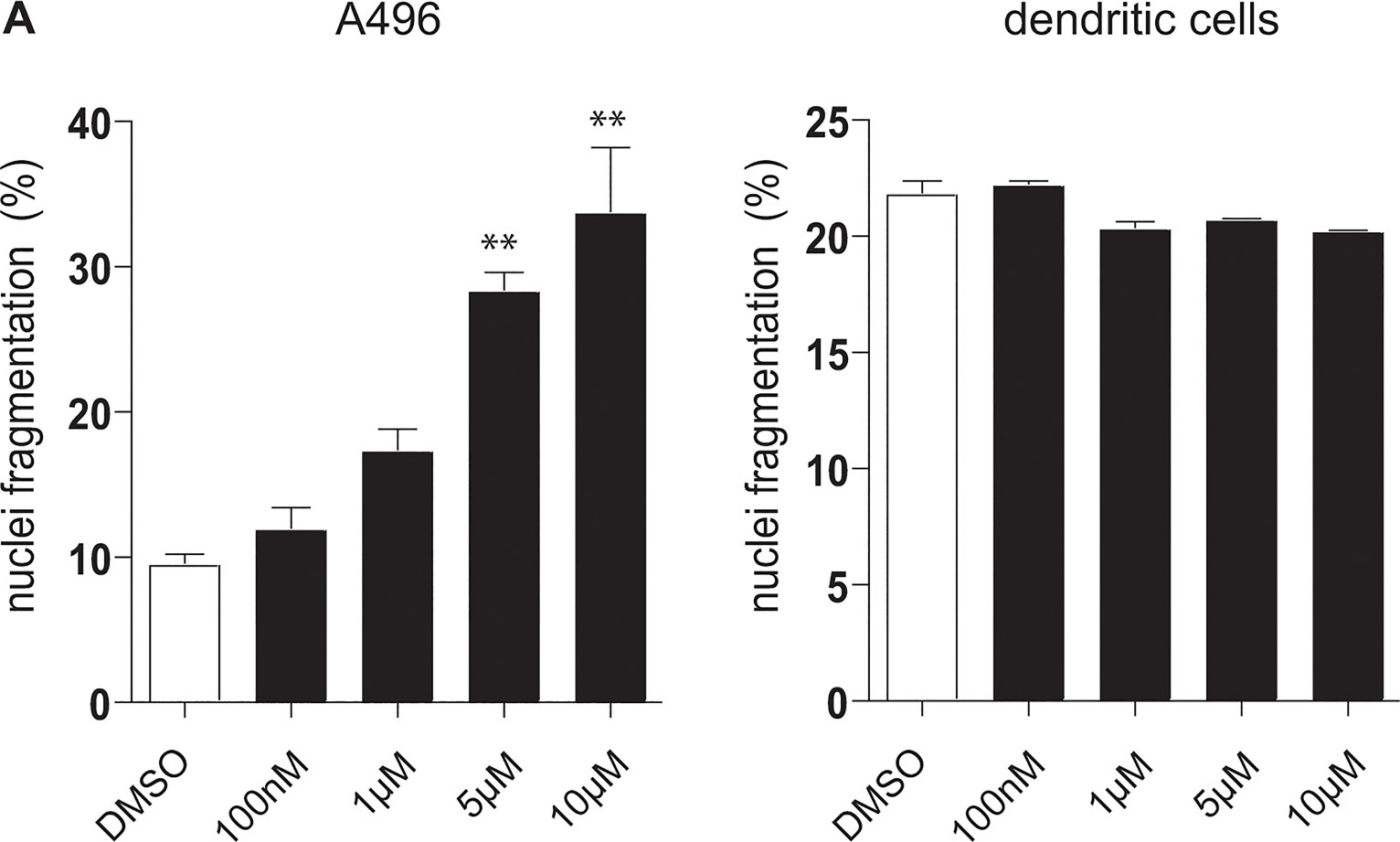
Axitinib dose-dependently induces apoptosis in the renal cell carcinoma cell line A-498, but has no toxic effects on dendritic cells. Different concentrations (100 nM– 10 μM; black columns) of axitinib or the vehicle control DMSO (white column) were added to the culture of monocyte-derived DCs generated in the presence of IL-4 and GM-CSF for 5 days (A) as well as to the culture of renal cell carcinoma cell line A-498 (B). Nuclei fragmentation was analyzed 48 hours later. Results of one representative donor out of 4 are shown.

We first tested the toxic effects of axitinib on monocyte-derived DCs (moDCs) generated in the presence of Interleukin-4 (IL-4) and granulocyte-macrophage colony-stimulating factor (GM-CSF) for 5 days. As a positive control, we analyzed the toxicity of axitinib on the renal cell carcinoma cell line A-498. Axitinib was added in increasing concentrations once to the culture and nuclei fragmentation was analyzed 48 hours later. While a significant amount of dose-dependent apoptosis induction was observed in the renal cell carcinoma cell line A-498 (Fig 1A), we did not detect a relevant induction of apoptosis by axitinib at doses up to 10 μM in moDCs (Fig 1B). This data was further confirmed by Western Blots analyzing caspase 3 expression (data not shown).

### Axitinib modulates moDC phenotype and decreases moDC activation

We next analyzed the effect of axitinib on phenotype and LPS-induced activation of moDCs generated in the presence of IL-4 and GM-CSF for 5 days.

**Fig 2:**
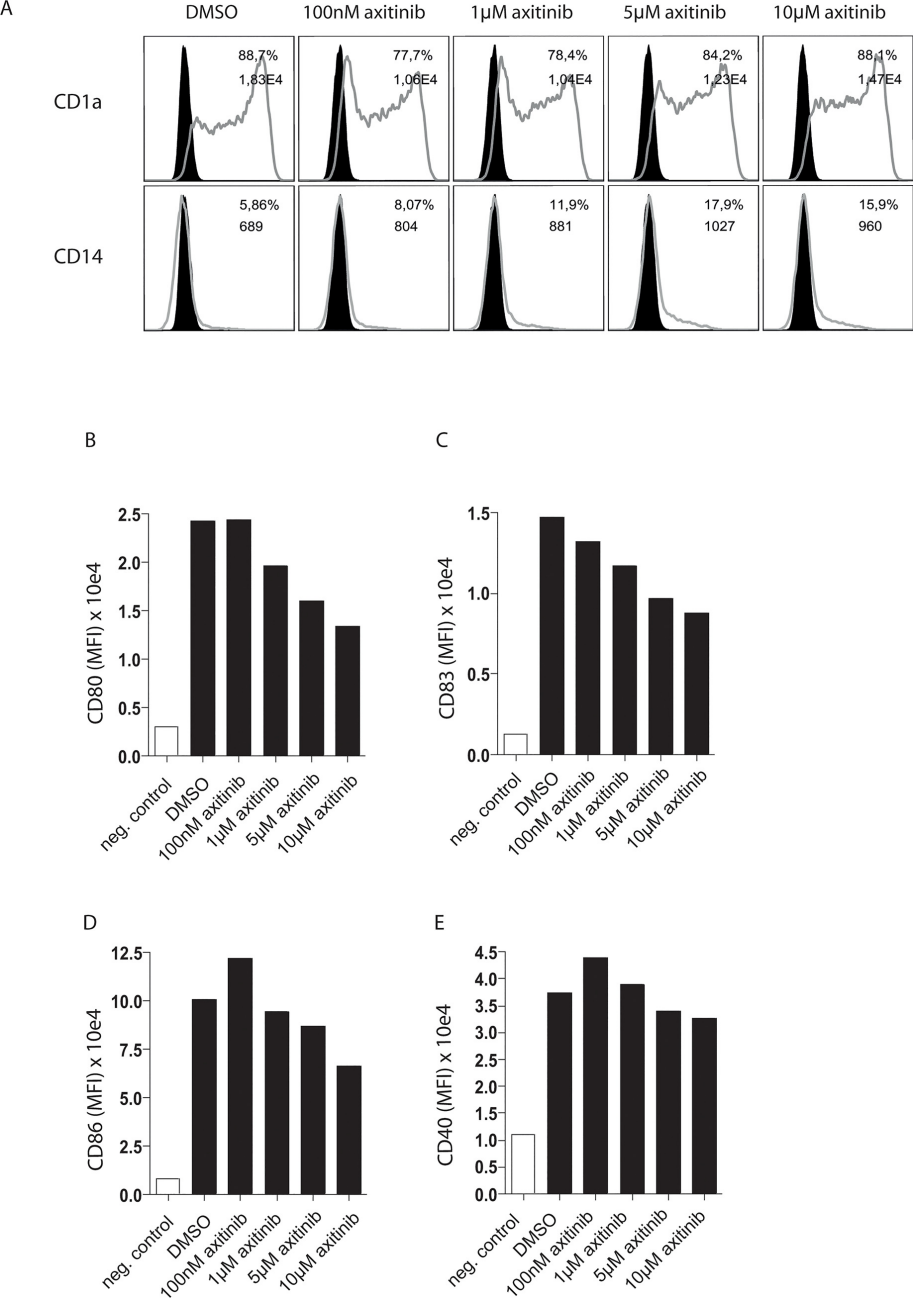
Axitinib modulates DC phenotype and DC activation. Expression of DC marker CD1a, monocyte marker CD14 and activation markers CD40, CD80, CD83 and CD86 after exposure of moDCs to axitinib (100 nM, 1 μM, 5 μM and 10 μM, black columns) on day 5 or the vehicle control DMSO (white column), followed by subsequent final maturation with LPS on day 6, are shown. Results of one representative donor out of 4 are shown.

A sole application of axitinib on day 5 did not modulate the expression of the bona fide DC marker CD1a, but re-induced expression of CD14 in a small proportion of the previously CD14 negative moDCs (CD14-expression 6% in DMSO- vs. 18% in 5 μM axitinib-treated moDCs; Fig 2A). More important, subsequent activation on day 6 revealed that axitinib dose-dependently impaired lipopolysaccharide (LPS)–induced up-regulation of the DC activation markers CD80, CD83 and CD86, while CD40 expression was not affected (Fig 2B–2E), in contrast to the vehicle control DMSO. This data indicates that axitinib interferes with moDC activation upon Toll-like receptor (TLR)-4-ligation.

### Axitinib modulates cytokine levels in moDCs

**Fig 3:**
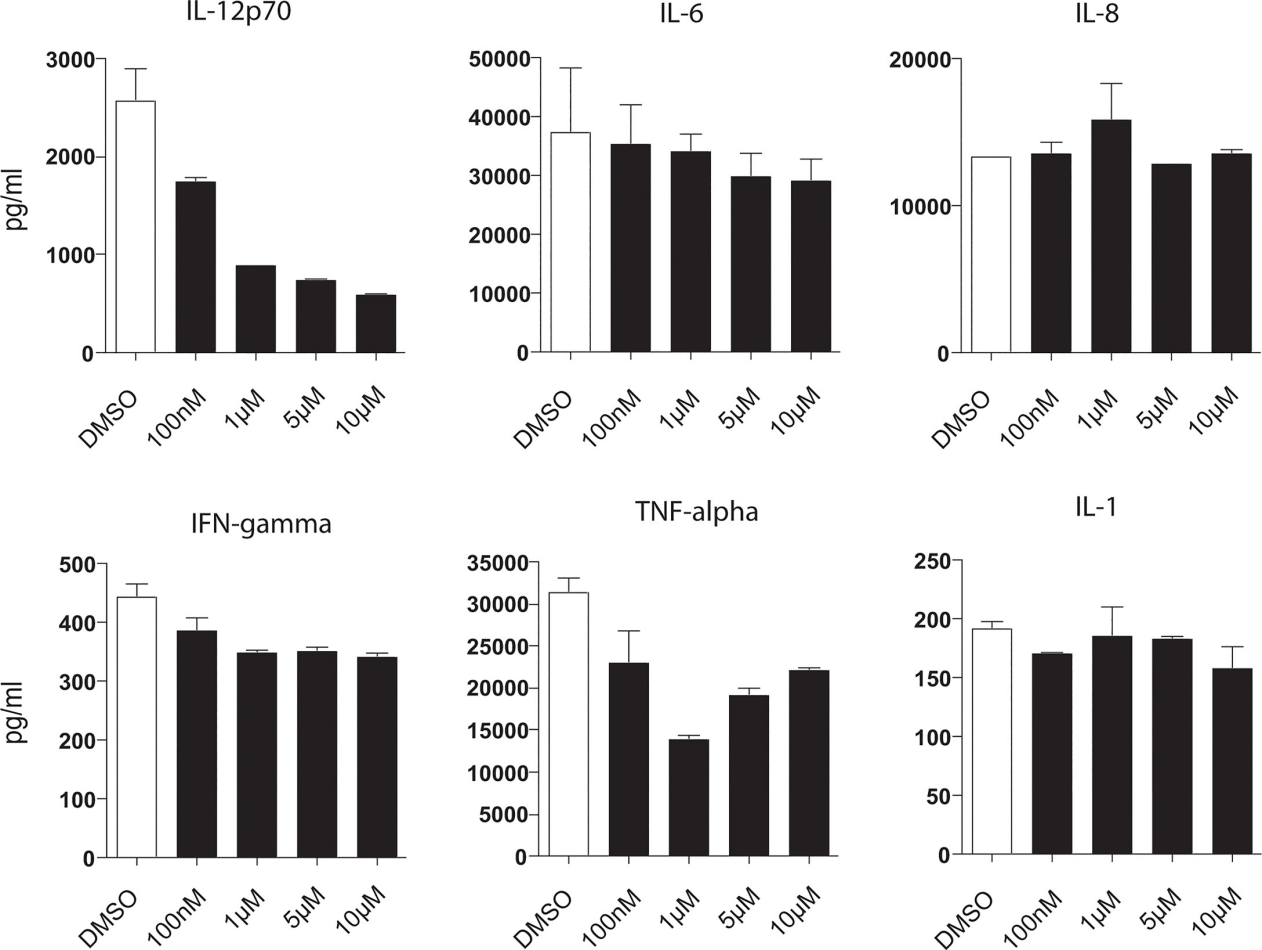
Axitinib modulates cytokine levels in DCs and impairs T cell stimulatory function of DCs. Monocytes were cultured under DC-driving conditions and treated with axitinib once on day 5, followed by LPS-activation on day 6. Supernatants were collected on day 7 and analyzed for cytokine production using a commercially available FlowCytoMix Assay. Results of one representative donor out of 3 are shown.

Cytokines are critical in the regulation of DC function as well as in their capacity to prime T cell responses. Thus we analyzed cytokine secretion in axitinib-treated human moDCs in response to TLR-4 stimulation. The most prominent reduction was detectable for interleukin-12 (IL-12; Fig 3A), which is critical for T cell activation, and tumor necrosis factor (TNF)-alpha. No significant modulation of cytokine responses was detected for interferon (IFN)-gamma, IL-6, IL-8 and IL-1β, underlining that a distinct effect and no general cytokine decrease is induced by axitinib.

### Axitinib reduces moDCs-mediated allogeneic T cell activation *in vitro*

**Fig 4:**
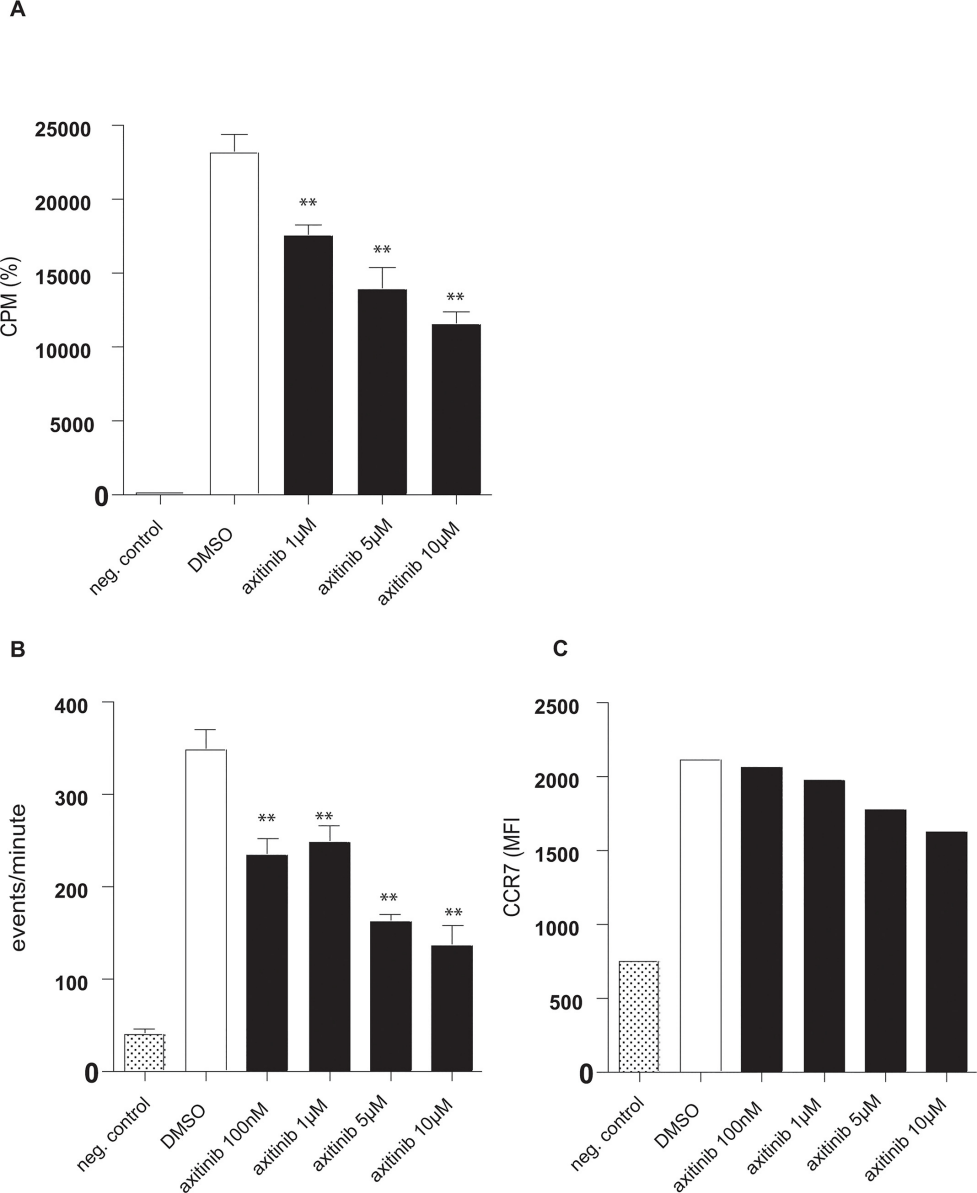
Axitinib impairs T cell stimulatory function and migratory behaviour of DCs. The ability of human moDCs, treated once with axitinib (black columns) on day 5 and activated with LPS on day 6, to prime allogeneic T cell responses *in vitro* was assessed using a mixed lymphocyte reaction assay. Irradiated stimulator DCs were cultured with responding allogeneic peripheral blood mononuclear cells. Tritium-labeled thymidine incorporation was measured 5 days later. White column represents DMSO as vehicle control, negative control represents CD3+ T cells without stimulating moDCs **(A)**. Axitinib-treated and LPS-stimulated human moDCs were assessed for their migratory behaviour towards CCL19/MIP-3β in transwell assays **(B)** and moDCs were analyzed for their surface CCR7 expression **(C).** White columns represent results of vehicle-exposed, LPS-stimulated DCs. Results are from one experiment representative of at least three. The significance was calculated according to one-way ANOVA Dunnett´s Multiple Comparison Test and is related to the vehicle control. **p<0.01.

Having shown that axitinib reduces DC activation and IL-12 production, we next assessed the ability of axitinib-pretreated human moDCs to prime allogeneic T cell responses *in vitro* using the mixed lymphocyte reaction (MLR). A sole application of axitinib on day 5 dose-dependently reduced TLR-4-activated moDC capacity to induce allogeneic T cell proliferation when compared to DMSO-treated controls (Fig 4A). Hence, axitinib reduces moDCs-mediated allogeneic T cell activation *in vitro*.

### Axitinib-treated moDCs show reduced migratory behavior

Mature moDCs express chemokine CC motif receptor 7 (CCR7), the receptor for CCL19/MIP-3β, which guides DC transit from peripheral tissue to draining local lymph nodes following a CCL19/MIP-3β gradient[7,13]. Migration of axitinib-treated and LPS-matured human moDCs towards MIP-3 was severely impaired (Fig 4B). Of note, flow cytometric analyses revealed a dose-dependent, but no significant reduction of LPS-induced CCR7 expression levels on moDCs, at least in part explaining the observed reduction of DC migration (Fig 4C).

### C-Rel is down-regulated in axitinib-treated moDCs

**Fig 5:**
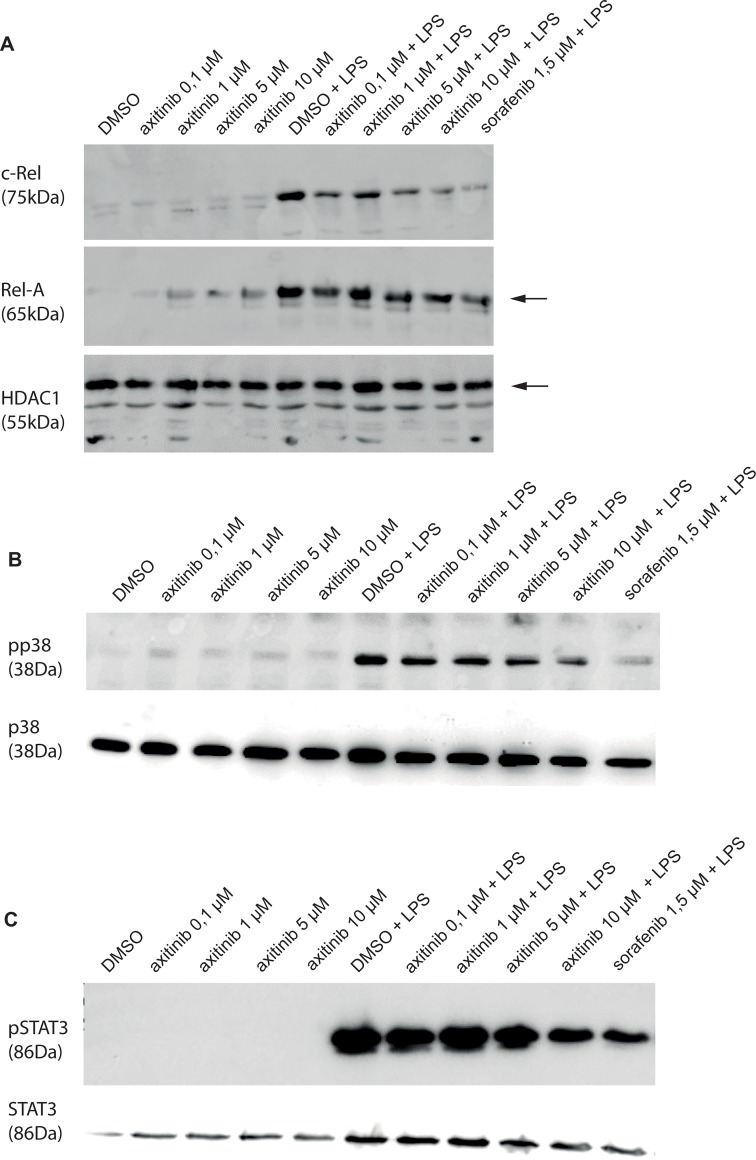
C-Rel, p38 and STAT3 are down-regulated in axitinib-treated moDCs. Whole cell lysates and nuclear extracts from axitinib or vehicle DMSO-treated moDCs, unstimulated or LPS-activated, were prepared and analyzed for their expression of RelA, cRel, p38, STAT3 in Western Blots. We found a dose-dependent down-regulation of cRel in axitinib-treated moDCs, whereas no significant modulation of RelA was observed in nuclear protein. The phosphorylation of p38 and STAT3 (measured in whole cell lysates) was dose-dependently decreased in higher doses, whereas the expression was not affected. HDAC1 was included as an additional loading control for nuclear protein expression. Results of one representative donor out of 3 are shown.

We next wanted to investigate the underlying mechanisms of DC modulation through axitinib. Nuclear proteins from axitinib or vehicle DMSO-treated moDCs were analyzed for their expression of RelA and c-Rel. C-Rel is known to establish a link between innate immune signals and primary T cell responses. The Rel/nuclear factor (NF)-kB signaling pathway is necessary for the induction of IL-12 in CD8+ DCs as major IL-12 producers[14,15]. We found a dose-dependent down-regulation of c-Rel in axitinib-treated LPS-activated moDCs which complement our data with an axitinib-induced decreased production of IL-12 by moDCs. In contrast, no significant modulation of RelA was observed (Fig 5A and S1 Fig).

### Axitinib-treated moDCs show reduced phosphorylation, but constant expression of p38 and STAT3

In order to further determine the underlying mechanism of DC dysfunction, we analyzed the MAPK and STAT signaling pathways in axitinib-treated moDCs. We found a dose-dependent reduced phosphorylation of p38 and STAT3, whereas the protein expression was not affected (Fig 5B and 5C and S2 and S3 Figs).

## Discussion

Angiogenesis and immunosuppression are tightly linked in tumor microenvironment. Tumor growth is associated with impaired antitumor immune responses, and is known to be closely associated with VEGF. VEGF and its receptor exert distinct effects on immune cells by modulating DC differentiation, DC trans-differentiation and DC-crosstalk[16,17]. Although therapies inhibiting VEGF receptors have demonstrated improved anti-tumor immunity and enhanced responses to cancer vaccines[4], DC modulation might interfere with the positive effects of VEGFR-inhibition. Sorafenib, but not sunitinib, has clearly been shown to modulate DC phenotype and function[4].

The VEGFR-inhibitor axitinib is an established second line treatment for metastatic RCC. However, not much is known whether axitinib affects DC phenotype and function and only limited mechanistic data on VEGFR-inhibitor- mediated immune-modulation is available.

We here show that axitinib affects human DC differentiation and function. We demonstrate that the addition of axitinib during the final lipopolysaccharide (LPS)-induced maturation step of monocyte-derived human DCs results in reduced DC activation. This was demonstrated by attenuated expression of the activation markers CD80, CD86 and, most profoundly, CD83. In addition, axitinib-treated moDCs produced significantly less interleukin (IL)-12, an observation that was further underlined by a dose-dependent down-regulation of c-Rel in axitinib-treated moDCs. IL-12 is mainly produced by APC and is essential for efficient T cell induction. Of note, it has been reported that maturation of DCs is not affected in the absence of c-Rel, whereas the loss of this protein in APC compromises DC-mediated T-cell activation[14]. Since we used DCs generated from human monocytes and no circulating human DCs in our experimental system, these moDCs might rather behave like the described APC. Hence, a reduction in c-Rel might indeed result in the observed attenuated IL-12 production. Furthermore, we found a dose-dependent reduced activation of p38 and STAT3, whereas the expression was not affected. This additionally underlines the axitinib-induced DC dysfunction.

Since T cells require efficient activation signals for successful T cell induction, we next analysed the induction of CD8+ T cell responses. Using mixed lymphocyte reaction assays, we were able to demonstrate that DC dysfunction was further underlined by their impaired induction of allogeneic T cell responses in vitro. Stehle and colleagues previously have performed a comparative analysis of sunitinib, sorafenib, and axitinib in regard to growth inhibitory properties and apoptosis induction on T cells and found all three TKIs to induce dramatic decreases in antigen-independent T cell proliferation[5]. The known sorafenib-mediated suppression of immune effector cells, in particular the reduction of the CD8+ T cell subset, was not observed in axitinib-treated immune effector cells in this study, which led the authors to the conclusion that axitinib, rather than sorafenib, seems to be the more suitable partner in complex treatment regimens of cancer patients including immunotherapy[5]. In contrast to our study, the colleagues did not focus on DCs and analysed T cell proliferation after stimulation with anti-CD3/anti-CD28 antibodies, while we aimed at investigating allogeneic DC-mediated CD8+ T cell induction. However, it is important to state that we agree that the effects of axitinib in regard to T cell proliferation seem to be less pronounced than the highly suppressive properties of sorafenib.

Due to the importance of proper DC migration to secondary lymphoid organs in order to induce T cell responses, we further focused on DC migration. VEGF signaling has already been associated with cell migration and also affects chemokine production. We found that axitinib-exposed, Toll-like receptor (TLR)-stimulated DCs exhibited a pronounced impairment of their migratory behaviour *in vitro*. This was, however, only partly due to a decreased induction of CCR7 on DCs which is in line with results from Stehle et al who have reported that axitinib only marginally affects C-C chemokine receptor type (CCR)7 expression on T cells[5]. DC migration is a multistep process that involves a huge variety of molecules. The exact mechanism of axitinib-induced reduced moDC migration is subject of ongoing studies.

Our results demonstrate that axitinib affects DC differentiation and function leading to impaired T cell activation. This provokes the question whether axitinib represents a useful combination partner of TKI treatment and immunotherapy—since attenuated DC activation is likely to interfere with a maximally warranted T cell induction immunotherapeutic approaches are aiming at. Axitinib has been investigated as such combination partner in only few pre-clinical trials. Combining axitinib with a specific peptide-based vaccination protocol using a subcutaneous B16.OVA melanoma mouse model yielded superior protection against melanoma growth and extended overall survival when compared with animals receiving either single modality therapy[18]. The anti-tumor effect in this study was postulated to be associated with a reduction in myeloid-derived suppressor cells (MDSCs) and regulatory T cells (Tregs) in the tumor, activation of tumor vascular endothelial cells, and activation and recruitment of vaccine-induced CD8+ T cells into the tumor. Also Zhang et al investigated the antitumor activity of axitinib against melanoma cells. The authors report that axitinib enhanced the proportion of CD8+ T cells and reduced the proportion of MDSCs in CD45.2 cells, whereas the proportions of CD4+ T cells and Tregs cells were not affected. Of note, axitinib suppressed the expression of pro-inflammatory cytokines such as IL-6, TNF-α, and IFN-γ[19].

Interestingly and in contrast to the anti-tumor efficacy of VEGFR inhibitors, it has also been reported that innate immune cell recruitment is suppressed by VEGFR inhibitors decreasing immune surveillance. This fact could help explain the increases in metastatic incidence and progression of tumors, recently linked to VGFR-inhibitors[16,20].

Taken together, axitinib decreases the building of new tumor vessels, reduces frequencies of MDSCs, maybe also Tregs and results in tumor shrinkage. We now add a further piece to the puzzle by showing that axitinib also modulates DC phenotype and function. Furthermore, T cell proliferation is reduced, although apparently less pronounced than with sorafenib treatment. Although the positive anti-tumor effects mediated by the VEGFR-inhibitor axitinib seem to outweigh the negative ones, the availability of different VEGFR-inhibitors and the specific knowledge of their distinct immune-modulatory effects requires the careful, attentive choice of each compound depending on the planned treatment plan. Our results may be of importance for the design of future vaccination protocols and combination regimens, especially in the context of immunotherapy with checkpoint inhibitors such as monoclonal antibodies against programmed death (PD)-1 which are currently tested in combination with TKIs.

## Supporting Information

S1 Fig C-Rel, but not Rel-A is downregulated in axitinib-treated moDCs. HDAC1 was included as an additional loading control for nuclear protein expression. The entire blot (of Fig 5A) is shown.
(EPS)

S2 Fig The phosphorylation of p38, but not its expression, is downregulated in axitinib-treated moDCs. The entire blot (of Fig 5B) is shown.
(EPS)

S3 Fig The phosphorylation of STAT3, but not its expression, is downregulated in axitinib-treated moDCs. The entire blot (of Fig 5C) is shown.
(EPS)
